# In vivo imaging of MmpL transporters reveals distinct subcellular locations for export of mycolic acids and non-essential trehalose polyphleates in the mycobacterial outer membrane

**DOI:** 10.1038/s41598-023-34315-4

**Published:** 2023-04-29

**Authors:** Laurie Thouvenel, Jérôme Rech, Christophe Guilhot, Jean-Yves Bouet, Christian Chalut

**Affiliations:** 1 Institut de Pharmacologie et de Biologie Structurale, Université de Toulouse, CNRS, UPS, Toulouse, France; 2 Laboratoire de Microbiologie et Génétique Moléculaires, Centre de Biologie Intégrative de Toulouse, CNRS, Université de Toulouse, UPS, Toulouse, France; 3 Present Address: de Duve Institute, UCLouvain, Brussels, Belgium

**Keywords:** Fluorescence imaging, Cellular microbiology, Membrane proteins, Glycolipids, Bacterial physiology

## Abstract

The mycobacterial cell envelope consists of a typical plasma membrane, surrounded by a complex cell wall and a lipid-rich outer membrane. The biogenesis of this multilayer structure is a tightly regulated process requiring the coordinated synthesis and assembly of all its constituents. Mycobacteria grow by polar extension and recent studies showed that cell envelope incorporation of mycolic acids, the major constituent of the cell wall and outer membrane, is coordinated with peptidoglycan biosynthesis at the cell poles. However, there is no information regarding the dynamics of incorporation of other families of outer membrane lipids during cell elongation and division. Here, we establish that the translocation of non-essential trehalose polyphleates (TPP) occurs at different subcellular locations than that of the essential mycolic acids. Using fluorescence microscopy approaches, we investigated the subcellular localization of MmpL3 and MmpL10, respectively involved in the export of mycolic acids and TPP, in growing cells and their colocalization with Wag31, a protein playing a critical role in regulating peptidoglycan biosynthesis in mycobacteria. We found that MmpL3, like Wag31, displays polar localization and preferential accumulation at the old pole whereas MmpL10 is more homogenously distributed in the plasma membrane and slightly accumulates at the new pole. These results led us to propose a model in which insertion of TPP and mycolic acids into the mycomembrane is spatially uncoupled.

## Introduction

Mycobacteria are endowed with a unique lipid-rich impermeable envelope composed of a plasma membrane, a cell wall consisting of a covalently linked complex of peptidoglycan, arabinogalactan and mycolic acids, and a capsule^1^. Mycolic acids bound to arabinogalactan interact with a wide variety of surface-exposed lipids (also known as free lipids) to form an outer membrane, called mycomembrane. The biogenesis of this multilayer dynamic structure is a tightly regulated process that requires the coordinated activities of multiple synthetic systems involved in the production of the cell envelope constituents^2^. Unlike other rod-shaped bacteria such as *E. coli* or *B. subtilis*, which grow through lateral-wall extension, mycobacteria elongate by incorporating new peptidoglycan at the cell poles^3,4^. This growth is asymmetric; the pole inherited from the mother cell (old pole) elongates more rapidly than the one arising from the most recent cell division (new pole)^3,5^. Many proteins involved in peptidoglycan biosynthesis during cell elongation and division are organized into large protein complexes, termed elongasome and divisome^3^. The subcellular localization and dynamic assembly of these complexes are controlled by dedicated proteins, including Wag31, which is responsible for the polar localization of the elongation complex and FtsZ, which plays a key role in divisome assembly^6^. Recent data showed that critical enzymes that control production of mycolic acids and arabinogalactan biosynthesis primarily localize with Wag31 to the cell poles, and particularly to the old pole, suggesting that synthesis of mycolic acids, arabinogalactan and peptidoglycan are closely coordinated during active growth in mycobacteria^7^. Moreover, visualization of the accumulation of newly synthesized mycolic acids at the cell poles during cell elongation by metabolic labeling experiments, further supports the notion that insertion of mycolic acids in the cell envelope occurs mainly at the cell poles^8–10^.

Despite these advances, many aspects of mycobacterial envelope assembly remain poorly understood. For instance, there is no information on the dynamics of incorporation of outer membrane lipids other than mycolic acids during cell elongation and division. In this study, we addressed this question by focusing on trehalose polyphleates (TPP), a family of outer membrane lipids produced by a wide range of mycobacterial species including *M. smegmatis*^11,12^. These lipids, composed of trehalose acylated with seven long-chain polyunsaturated fatty acids and a C14-C18 straight fatty acid residue, share structural features, as well as conserved production and export mechanisms, with polyacyl trehaloses and sulfolipids, two major classes of outer membrane glycolipids specific to *M tuberculosis*^13^. In contrast to mycolic acids, but like polyacyl trehaloses and sulfolipids in *M. tuberculosis*, TPP are dispensable for bacterial growth^11^. Therefore, studying the dynamics of TPP incorporation into the outer membrane of *M. smegmatis* could provide valuable information on cell envelope biogenesis in *M. tuberculosis* during cell growth. Here, we investigated whether export of TPP is coordinated with mycolic acid export in actively growing cells by examining the cellular locations of MmpL3, MmpL10 and Wag31. MmpL3 mediates the transfer of mycolic acids through the form of trehalose monomycolate (TMM) across the plasma membrane and its location into the cell envelope coincides with the sites of incorporation of newly synthesized mycolic acids in the outer membrane^14–16^. MmpL10 is involved in the transport of TPP intermediates through the inner membrane^11,12^. We postulated that, like MmpL3, its location in the inner membrane coincides with or is in close proximity to the sites of incorporation of newly synthesized lipids in the outer membrane. Indeed, given their extreme hydrophobicity, outer membrane lipids are not expected to reside in the periplasmic space after translocation across the plasma membrane but to be promptly transferred into the mycomembrane. Using fluorescence microscopy approaches, we found that the two transporters display different spatial distributions in *M. smegmatis*, and that unlike MmpL3, MmpL10 does not localize with Wag31 along the cell cycle. These results show a disconnection between incorporation of TPP and mycolic acids into the cell envelope and shed new light on the coordination between outer membrane assembly and cell wall synthesis.

## Results

### MmpL3 and MmpL10 have distinct subcellular localization patterns

**Figure 1:**
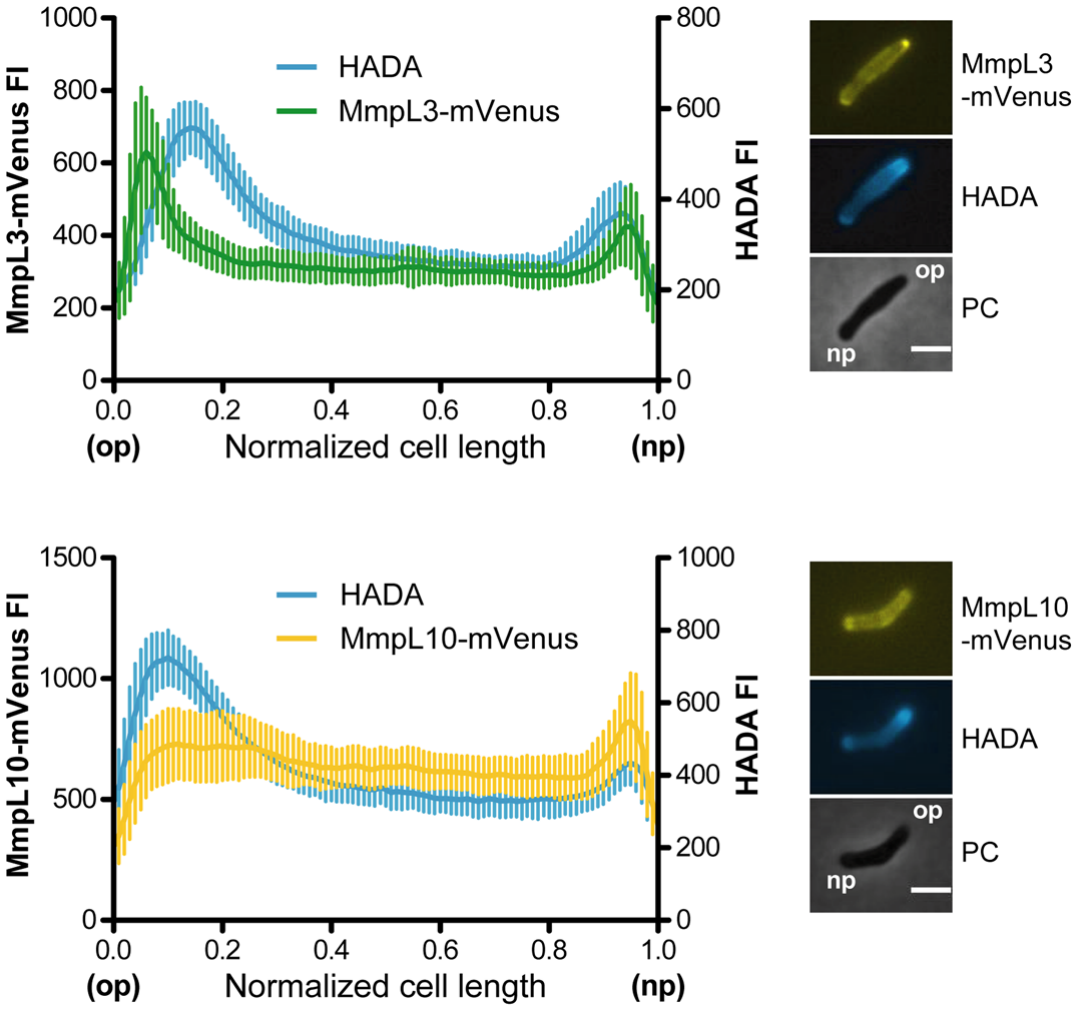
Fluorescence intensities (FI) of MmpL3-mVenus and MmpL10-mVenus over the long axis of *M. smegmatis* cells pulse-labeled with HADA. Plot profiles represent average fluorescence intensities (arbitrary units) as a function of the normalized distance to the old pole of each cell, calculated on images obtained from bacteria expressing either MmpL3-mVenus (WT/pNL3mVen, upper) (n = 20) or MmpL10-mVenus (PMM223/pNL10mVen, lower) (n = 27) labeled with HADA for 20 min. Cell poles with brighter HADA fluorescence correspond to old poles. Vertical lines represent the standard deviations of the mean fluorescence at each position along the cell axis. The graphs are representative of at least three independent experiments. Representative images of *M. smegmatis* cells expressing MmpL10-mVenus or MmpL3-mVenus, pulse-labeled with HADA are shown on the left (Scale bar, 2 μm). *PC* phase contrast, *op* old pole, *np* new pole.

To investigate the cellular distribution of MmpL3 and MmpL10 in *M. smegmatis*, we expressed either MmpL3 or MmpL10, fused at their C-termini to the yellow fluorescent protein mVenus^17^ (Table 1). The MmpL10-mVenus protein was produced from a fusion gene under the control of the native *mmpL10* promoter in PMM223, a *M. smegmatis* strain impaired in TPP production containing an unmarked deletion mutation into *mmpL10*, transformed with plasmid pNL10mVen (Supplementary Fig. S1 online and Table 1). In contrast the MmpL3-mVenus protein was expressed in the wild type strain of *M. smegmatis* after transformation of bacteria with pNL3mVen (Table 1) because of the essentiality of the *mmpL3* gene, which makes it not possible to generate an *mmpL3* knockout mutant^14^. Western blot analysis of total crude extracts prepared from the WT/pNL3mVen and PMM223/pNL10mVen strains showed that the fluorescent fusion proteins underwent only minor degradation in growing cultures with, however, a greater susceptibility of MmpL10-mVenus to proteolytic cleavage (Supplementary Fig. S2 online). Furthermore, these two strains did not show any morphological or growth changes and produced similar amounts of TMM/TDM and TPP compared to the *M. smegmatis* wild type strain (Supplementary Fig. S3 online). Importantly, restoration of TPP production in the PMM223/pNL10mVen strain indicated that the MmpL10-mVenus protein was correctly folded and functional. We then performed snapshot fluorescence imaging of *M. smegmatis* cells expressing either MmpL3 or MmpL10 fluorescent proteins. To distinguish between the old and new poles, bacterial cells were pulse-labeled with 7-Hydroxycoumarin-amino-D-alanine (HADA), a fluorescent derivative of D-alanine that is incorporated into peptidoglycan at sites of active synthesis^18^, and fluorescence intensities of MmpL3 and MmpL10 fusion proteins were measured along the longitudinal axis of the cells (Fig. 1). Fluorescence profiles were normalized relative both to cell length and old-new pole orientation. Multiple analyses of independent cells expressing either MmpL3-mVenus (top panel) or MmpL10-mVenus (bottom panel) revealed markedly different distribution patterns of the transporters in bacteria. MmpL3-mVenus localized at the two cell poles with a strong preference for the old pole, as reported in previous studies^7,16^. By contrast, MmpL10-mVenus displayed a more homogenous distribution in the cell envelope with slight accumulation at the new pole. These observations suggest that the subcellular localization of these transporters is governed by distinct mechanisms.

**Table 1:** Strains and plasmids.

Name	Relevant characteristics	Ref./source
Strains		
WT	*M. smegmatis* strain mc^2^155	–
WT/pNL3mVen	WT expressing MmpL3-mVenus	This study
WT/pNL3Wag	WT expressing MmpL3-mVenus and Wag31-mCherry	This study
PMM223	*M. smegmatis* mc^2^155 Δ*mmpL10::res*	This study
PMM223/pNL10mVen	PMM223 expressing MmpL10-mVenus	This study
PMM223/pNL10Wag	PMM223 expressing MmpL10-mVenus and Wag31-mCherry	This study
Plasmids		
pNL3mVen	pMV361eH containing *mmpL3* fused to the *mVenus* gene at the 3’ end, under the control of the *mmpL10* promoter, Hyg^R^	This study
pNL10mVen	pMV361eH containing *mmpL10* fused to the *mVenus* gene at the 3’ end, under the control of its own promoter, Hyg^R^	This study
pNL3Wag	pMV361eH containing *mmpL3* and *wag31* respectively fused to the *mVenus* and *mCherry* genes, at the 3’ends, Hyg^R^	This study
pNL10Wag	pMV361eH containing *mmpL10* and *wag31* respectively fused to the *mVenus* and *mCherry* genes, at the 3’ends, Hyg^R^	This study
pMVL10mVen	pMV361eH containing *mmpL10* behind the pblaF* fused to the *mVenus* gene at the 3’ end, Hyg^R^	This study

### MmpL3 co-localizes with Wag31 during the cell cycle but not MmpL10

**Figure 2:**
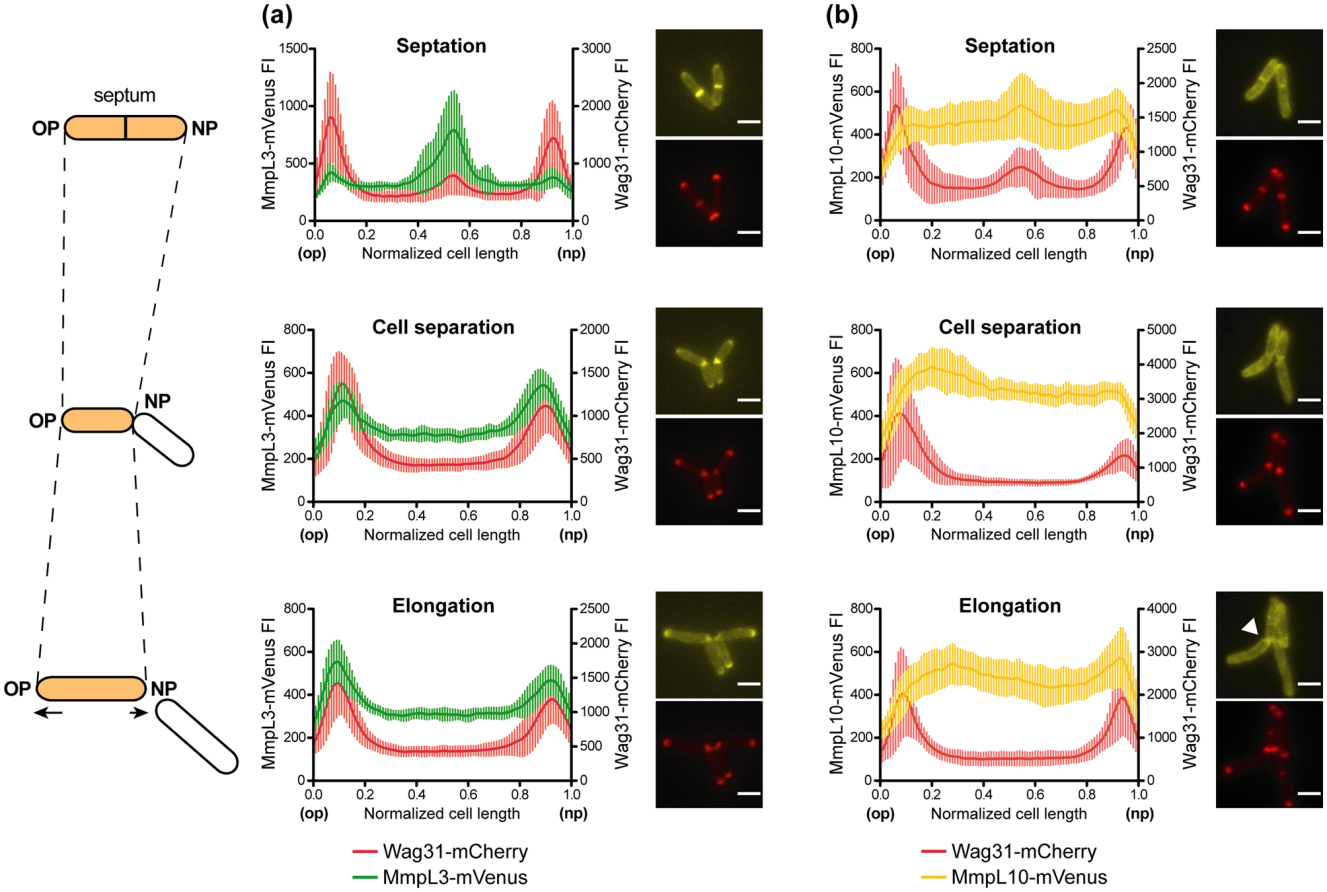
Fluorescence intensities of MmpL3-mVenus, MmpL10-mVenus and Wag31-mCherry over the long axis of *M. smegmatis* cells. Plot profiles are represented as in Fig. 1 and calculated on snapshot images obtained from bacteria that expressed either MmpL3-mVenus in combination with Wag31-mCherry (WT/pNL3Wag, panel (**a**) or MmpL10-mVenus in combination with Wag31-mCherry (PMM223/pNL10Wag, panel (**b**) at three stages of the mycobacterial cell cycle i.e. septation, cell separation (V-snapping), elongation. For each stage of the cell cycle: n > 15 for WT/pNL3Wag and n > 10 for PMM223/pNL10Wag. Vertical lines represent the standard deviations of the mean fluorescence at each position along the cell axis. Representative images of *M. smegmatis* cells expressing either MmpL3-mVenus/Wag31-mCherry or MmpL10-mVenus/Wag31-mCherry are shown on the left of graphs for each stage of the cell cycle (Scale bar, 2 μm). *op* old pole, *np* new pole. The white arrowhead shows the accumulation of MmpL10-mVenus at the new cell poles after cell separation.

We then investigated the subcellular localization of MmpL10 and MmpL3 during cell cycle progression by time-lapse fluorescence microscopy, using Wag31 as a cell cycle marker. This protein regulates cell elongation by acting as a scaffold for cell wall synthesis proteins at the cell poles and accumulates at the septum during cytokinesis before daughter cell separation^19^. Two vectors expressing a mCherry-tagged Wag31 protein in combination with either MmpL3-mVenus (pNL3Wag) or MmpL10-mVenus (pNL10Wag) were generated and transferred into the WT strain of *M. smegmatis* or PMM223, respectively (Table 1). In these bacteria, the Wag31-mCherry fusion was produced in the presence of the native untagged Wag31 to prevent ectopic localisation of the fusion protein at the new pole and abnormal cell morphology, as previously reported by Meniche et al*.*^7^. The WT/pNL3Wag and PMM223/pNL10Wag strains exhibited similar growth rate, cell size and lipid profiles to the wild-type strain (Supplementary Fig. S3 online). For each strain, we monitored the distribution and dynamics of the fluorescent MmpL and Wag31 proteins at the single-cell level over several cell cycles. Snapshot images representative of three stages of the mycobacterial cell cycle, i.e. septation, cell separation (V-snapping), elongation, were selected and fluorescent intensities of fusion proteins were quantified along the cells and their distribution normalized to cell size oriented from old to new pole. As expected, in both WT/pNL3Wag and PMM223/pNL10Wag, Wag31 accumulated at the cell poles at each stage of the bacterial cell cycle, with a preference for the old pole, and was present at the septum only during cell division (Fig. 2 and Supplementary Movies S1 and S3 online). MmpL3-mVenus displayed a distribution profile similar to that of Wag31 (Fig. 2 and Supplementary Movies S1 and S2 online). At the division stage, MmpL3-mVenus exhibited a strong fluorescence spot at mid-cell, indicating that the transporter is recruited at the septum. At the cell separation stage (V-snapping), MmpL3 localized with Wag31 at the cellular poles where it remained during the elongation stage.

MmpL10-mVenus was more prominently present in the lateral region of the cell envelope and exhibited a different distribution pattern at the poles during cell cycle. The fusion protein showed fluorescence signals at the septum during the division stage (Fig. 2 and Supplementary Movies S3 and S4). However, this signal was weaker compared to MmpL3. This could be related to the presence of two distinct inner membranes at the division site during septation, resulting in a twofold increase in the local concentration of MmpL10-mVenus. This hypothesis is supported by the loss of the fluorescence signal during V-snapping. Remarkably, MmpL10-mVenus concentrated at the new cell poles after separation of daughter cells (Fig. 2). As cells elongated, we observed a redistribution of the fluorescent signal towards the lateral zone of the cell envelope, reflecting either a relocalization of the transporter to the sidewall or an accumulation of de novo synthesized transporter in this area, along with a degradation of pole-associated MmpL10 proteins. We noticed that increasing the production of MmpL10-mVenus in bacteria, through the use of an alternative plasmid harboring *mmpL10* under the control of the strong pBlaF* promoter (pMVL10mVen, Table1), resulted in higher fluorescence signals but did not change its localization pattern, indicating that modulating the expression level, at least in the range used in this study, does not alter the behavior of the protein (Supplementary Movie S5 online). Altogether, our data show that MmpL10-mVenus and Wag31 differ widely in their cellular localization during cell cycle progression, revealing that unlike mycolic acids, insertion of TPP into the cell envelope is spatially dissociated from peptidoglycan assembly.

## Discussion

The aim of this study was to provide information on the assembly of the mycobacterial cell envelope, and more specifically on the dynamics of incorporation of lipids in the mycomembrane during cell growth. We focused on two families of outer membrane lipids, namely mycolic acids and TPP, and used MmpL3 and MmpL10 to visualize the localization of the translocation of these lipids from the cytoplasm to the cell envelope. These transporters mediate periplasmic export of mycolic acids and TPP intermediates and their distribution in the plasma membrane probably dictates the location of lipid insertion sites in the outer membrane, as previously shown for MmpL3 and mycolic acids^15,16^. We established that the two transporters exhibit markedly different dynamic behaviors. In agreement with previously published data, we found that MmpL3 remains colocalized with Wag31 at the cell poles, mainly the old pole, and septum throughout the cell cycle^7,16^. In sharp contrast, we observed an accumulation of MmpL10 at the new pole after cell division and a localization of the transporter in the sidewall during the elongation phase. The unchanged growth rate and the identical cell envelope lipid composition of our recombinant strains compared to the wild type strain indicate that the physiological state of the bacteria is not affected by the expression of the MmpL fusion proteins.

We cannot exclude that common issues arising from the use of fluorescent tagged proteins may affect our data analyses. For instance, the fluorescent tag might affect membrane topology or interactions with potential MmpL partners, leading to altered subcellular localization of fusion proteins. In addition, our Western blot analyzes revealed a probable relative overexpression of fusion proteins compared to endogenous proteins, as well as partial protein cleavage, which may result in protein mislocalization or aggregation. In this regard, the MmpL10-mVenus protein seemed to be expressed at a higher level than MmpL3-mVenus in bacteria and be more prone to proteolytic cleavage. Nevertheless, TPP production and transport appeared identical in strains expressing the MmpL10-mVenus compared to the WT strain, supporting that MmpL10 was functional and that possible artefacts do not significantly alter the conclusions of our imaging data. Indeed truncated proteins are usually rapidly degraded or lose their ability to localize to specific sites in cells, leading to a diffuse distribution of the fluorescent signal. Alternatively, if aggregation occurs, it would accumulate at the cell poles and remain static throughout the cell cycle^20^. This contrasts sharply with the behavior of our fusion proteins. The fluorescence signal was detected primarily in the cell envelope, consistent with the subcellular location of the MmpL3 and MmpL10 transporters, and more importantly we observed a dynamic and specific fluorescence labeling pattern over the course of the cell cycle, a reliable indicator of non-artifactual localization of proteins.

Our findings support a model of cell envelope assembly in which incorporation of mycolic acids and TPP are spatially disconnected. Colocalization between MmpL3 and Wag31 provides strong evidence that peptidoglycan assembly and insertion of mycolic acids into the cell envelope occur together at cell poles and septum during cell elongation and division. This scenario is consistent with the structural role of mycolic acids in the architecture of the cell envelope which, in addition to being present as free lipids in the form of TMM and TDM in the outer membrane, are essential components of the mycobacterial cell wall. It is reasonable to assume that assembly of the different constituents of this cell wall is coordinated in space and time to ensure cell envelope integrity by regulatory mechanisms that remain to be clarified. In this regard, it has been reported that MmpL3 interacts with Wag31 and with several proteins involved in cell elongation and septation such as CrgA, a cell division regulatory protein^21,22^. Likewise, a membrane protein, namely TtfA, was found to play a crucial role in the cellular location of MmpL3 but the connections, if any, between this protein and the elongation and division machineries are unknown^23^.

The presence of substantial amounts of MmpL10 in the lateral cell envelope during bacterial elongation suggests that, unlike mycolic acids, TPP export occurs predominantly all along the periphery rather than at the cell poles and is spatially decoupled from cell wall biogenesis. Interestingly, MmpL10 accumulates at the new pole in newborn cells. Recently it has been proposed that mycobacteria display a NETO (new end take off) growth model in which the new pole initiates growth after a delay^5^. After a lag phase, the new cell pole switches from slow to fast growth and elongates at a rate similar to that of the old pole. It is conceivable that MmpL10 accumulates at the new pole during the lag phase by interacting with as yet unknown partner(s) and is released when the elongation complex is recruited to initiate growth.

The insertion of mycolic acids and TPP at different locations in the outer membrane raises the question of lateral lipid heterogeneity in this structure. Metabolic labeling of *M. smegmatis* or *M. tuberculosis* with trehalose analogues revealed that mycolic acids are incorporated at the cell poles and move to the sidewall as cell elongation progresses, resulting in a uniform distribution of mycolic acids throughout the cell envelope^8–10,24^. The dynamic of incorporation and spatial distribution of other outer membrane lipids have never been investigated because of the lack of specific metabolic markers to track their cellular localization but our data highlighting the uniform distribution of MmpL10 in the lateral cell envelope suggest that TPP are inserted in the sidewall and are evenly distributed within the outer membrane. Therefore, the different cellular locations of MmpL3 and MmpL10 point to distinct integration sites for mycolic acids and TPP without necessarily leading to a heterogeneous and distinct distribution of these lipids in the cell envelope.

To refine our model of cell envelope assembly, it would be interesting to examine the cellular distribution of MmpL involved in the transport of non-essential outer membrane lipids other than TPP in *M. smegmatis*, such as glycopeptidolipids, to determine if these lipids are also exported uniformly or at specific sites in the cell envelope^25^. Finally, the conclusions drawn from this study on the assembly of the *M. smegmatis* cell envelope can probably be extended to *M. tuberculosis*. Indeed, polyacyl trehaloses and sulfolipids, two major classes of glycolipids found in the outer membrane of *M. tuberculosis*, share structural characteristics with TPP^13^. These lipids are dispensable for bacterial growth in vitro and exhibit conserved mechanisms of production and export with TPP, raising the possibility that their incorporation into the cell envelope is spatially disconnected from that of mycolates during cell cycle progression in *M. tuberculosis*.

## Methods

### Bacterial strains, growth media, and culture conditions

Plasmids were propagated at 37 °C in *E. coli* DH5α in LB broth or LB agar (Invitrogen) supplemented with either kanamycin (Km) (40 µg/ml) or hygromycin (Hyg) (200 µg/ml). *M. smegmatis* mc^2^155 WT and derivatives (Table 1) were grown at 37 °C in Middlebrook 7H9 broth (DB Difco) containing ADC (0.2% dextrose, 0.5% BSA fraction V, 0.0003% beef catalase) and 0.05% Tween 80 when necessary and on LB agar solid medium. When required, Km and Hyg were used at a concentration of 40 and 50 µg/ml, respectively.

### Construction of strains and plasmids

The strategy for the construction of the PMM223 mutant strain is depicted in Supplementary Fig. S1. Briefly, two DNA fragments encompassing the regions located upstream and downstream of the *mmpL10* gene (*MSMEG_0410*) were amplified by PCR from *M. smegmatis* mc^2^155 total DNA and cloned, flanking a *res*-*km*-*res* resistance cassette into the mycobacterial suicide plasmid pJQ200 bearing the counterselectable marker *sacB* gene^26,27^. The resulting plasmid was transferred by electroporation into *M. smegmatis* mc^2^155 for allelic exchange and transformants were selected on LB agar plates containing Km and sucrose. Several transformants were isolated and analyzed by PCR using combinations of primers located outside of the regions of homology (23016a and 23016b) and within the *res*-*km*-*res* resistance cassette (res1 and res2) or the deleted region of *mmpL10* (23016c). One mutant with the correct deletion was selected and named PMM222 (Δ*mmpL10*::*res-km-res*). To generate PMM223, the *res*-*km*-*res* cassette inserted in the *mmpL10* gene of PMM222 was recovered by site-specific recombination between the two *res* sites using the thermosensitive plasmid pWM19 that contains the resolvase gene of transposon γδ, as previously described^11,26^. Genomic DNA from PMM223 was isolated and analysed by PCR with primers used for the characterization of PMM222.

To construct pMVL10mVen, the *mmpL10* gene without its stop codon was first amplified by PCR from *M. smegmatis* mc^2^155 total DNA using primers 16165a and 16165c (Supplementary Table S1) and cloned between the *Nde*I and *Hind*III restriction sites of pMV361eH, a pMV361 derivative containing the pblaF* promoter and carrying a *hyg* resistance marker^11^. The *mVenus* gene was amplified by PCR from plasmid pJYB318^28^ using primers 17331a and 17331b (Supplementary Table S1) and then inserted by In-Fusion cloning (Clontech) downstream of the *mmpL10* gene. To construct pNL10mVen, the upstream region of the *papA3*-*mmpL10* operon^11^ was amplified by PCR from *M. smegmatis* total DNA using primers 18075d and 18075e (Supplementary Table S1) and cloned between the *Pac*I and *Nde*I restriction sites of pMVL10mVen, in front of *mmpL10* to replace the original pBlaF* promoter. To construct pNL3mVen, the *mmpL3* gene (*MSMEG_0250*) was amplified by PCR from *M. smegmatis* mc^2^155 total DNA using primers 19170a and 19170b (Supplementary Table S1) and cloned between the *Nde*I and *Hind*III restriction sites of pNL10mVen, to replace the *mmpL10* gene. To generate pNL10Wag and pNL3Wag, a DNA sequence overlapping the *wag31* gene (*MSMEG_4217*) plus an upstream sequence was PCR-amplified from *M. smegmatis* genomic DNA and inserted upstream of the *mCherry* gene cloned in pMV361e. The *wag31-mCherry* fusion DNA sequence was then amplified with 19318a and 19318b (Supplementary Table S1) and cloned in the *Xba*I restriction site of either pNL10mVen or pNL3mVen.

### Fluorescence microscopy technics

Bacteria from a 2-day-old preculture were diluted 500 times in fresh 7H9 medium supplemented with 0.05% Tween 80 and grown for approximatively 12 h at 37 °C in a shaking incubator, until reaching an OD_600_ nm of 0.3. The cultures were then incubated with 500 µM of HADA (7-Hydroxycoumarin-amino-d-alanine, Bio-Techne) in DMSO for 20 min at 37 °C with shaking in the dark, then washed twice in 7H9/Tween 80. 0.6 µl of each culture was then spotted on 1% agarose pads in 7H9. Bacteria were visualized with an Eclipse TI-E/B bright-field fluorescence microscope equipped with a phase contrast objective (CFI Plan Apo LBDA 100X oil NA 1.45) and Semrock filters: CFP (excitation: 438BP24; dichroic mirror: 458; emission: 483BP32), YFP (excitation: 500BP24; dichroic mirror: 520; emission: 542BP27) and mCherry (excitation: 562BP40; dichroic mirror: 593; emission: 641BP75). Images were acquired with a Neo SCC-02124 Andor camera using 20 to 75% illuminations from SpectraX Led sources (Lumencor) and using exposure times ranging from 200 ms to 1 s. Images were taken using the Nis-Elements AR software (Nikon).

### Normalisation of fluorescence signals

Fluorescence microscopy images were analysed using the Fiji ImageJ software (the MultiStackReg plugin was used on timelapse movies). Each bacterium lacking a visible septum was manually picked and oriented according to the HADA marker, from the brightest pole (old pole) to the fainter pole (new pole). A segmented line was drawn from the old pole to the new pole, through the longitudinal axis of each bacterium. Fluorescence intensities were extracted on each line and plotted against normalized cell length using an in-house software program.

## Supplementary Information

Supplementary Information.

Supplementary Video 1.

Supplementary Video 2.

Supplementary Video 3.

Supplementary Video 4.

Supplementary Video 5.

## Data Availability

The data used to support the findings of this study are available from the corresponding author upon request.
