# Supplementary material for: In vivo imaging of MmpL transporters reveals distinct subcellular locations for export of mycolic acids and non-essential trehalose polyphleates in the mycobacterial outer membrane
Source: Sci Rep. 2023 Apr 29;13:7045. doi: 10.1038/s41598-023-34315-4 (PMC10148836; doi:10.1038/s41598-023-34315-4)
Supplement: Supplementary file 1 — Supplementary Information. [file 41598_2023_34315_MOESM1_ESM.pdf]

Supplementary information

In vivo imaging of MmpL transporters reveals distinct subcellular locations for export of mycolic acids and non-essential trehalose polyphleates in the mycobacterial outer membrane

Laurie Thouvenel[1,a], Jérôme Rech[2], Christophe Guilhot[1], Jean-Yves Bouet[2], and Christian Chalut[1,*].

List of the material included

- **Supplementary Figure S1**. Generation and analysis of the PMM223 mutant strain.

- **Supplementary Figure S2.** Western blotting analysis of total cell lysates from strains used in this study.

- **Supplementary Figure S3.** Growth curves, cell length measurements and lipid profiles of strains used in this study.

- **Supplementary Table S1.** Oligonucleotides used in this study.

- **Supplementary Videos S1 and S2** (provided online). Timelapse movies of WT/pNL3Wag showing Wag31-mCherry localization (video S1) and MmpL3-mVenus localization (video S2).

- **Supplementary Videos S3 and S4** (provided online). Timelapse movies of PMM223/pNL10Wag showing Wag31-mCherry localization (video S3) and MmpL10-mVenus localization (video S4).

- **Supplementary Video S5** (provided online). Timelapse movie of PMM223/pMVL10mVen expressing MmpL10-mVenus.

- **Supplementary References.**

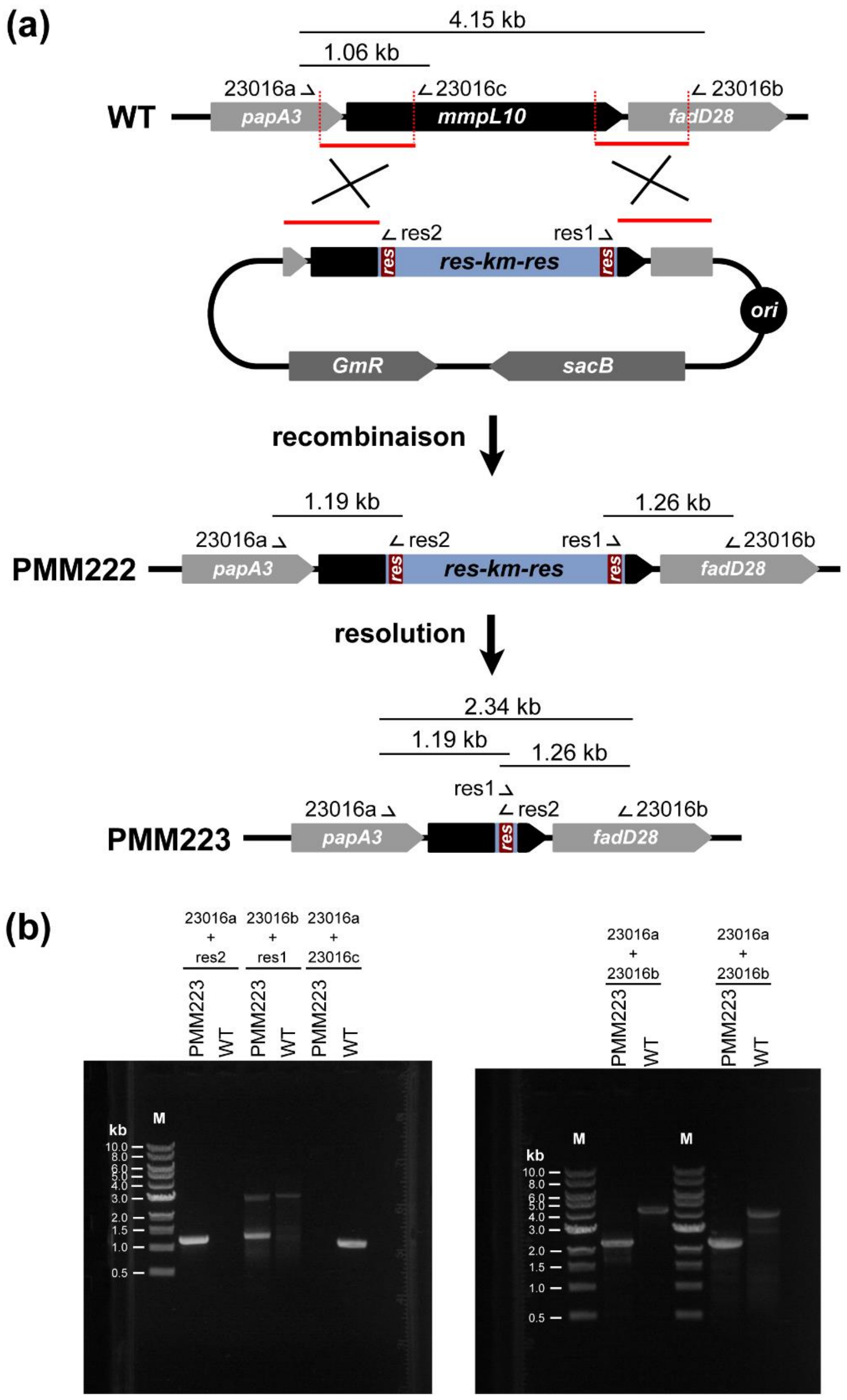

Supplementary Figure S1: Generation and analysis of the PMM223 mutant strain. **(a)** Cloning strategy for the construction of PMM223 (for details see methods). The two DNA fragments upstream and downstream of the *mmpL10* gene used for the construction of the allelic exchange substrate are represented by red lines. **(b)** PCR analysis of the PMM223 mutant strain. Genomic DNA from *M. smegmatis* WT and PMM223 were isolated and analyzed by PCR using combinations of primers located outside of the regions of homology and within the sequence *res*. Positions of primers and the predicted sizes of PCR products are shown above each genetic structure. Primer sequences are shown in the Supplementary Table S1. kb, kilobase.

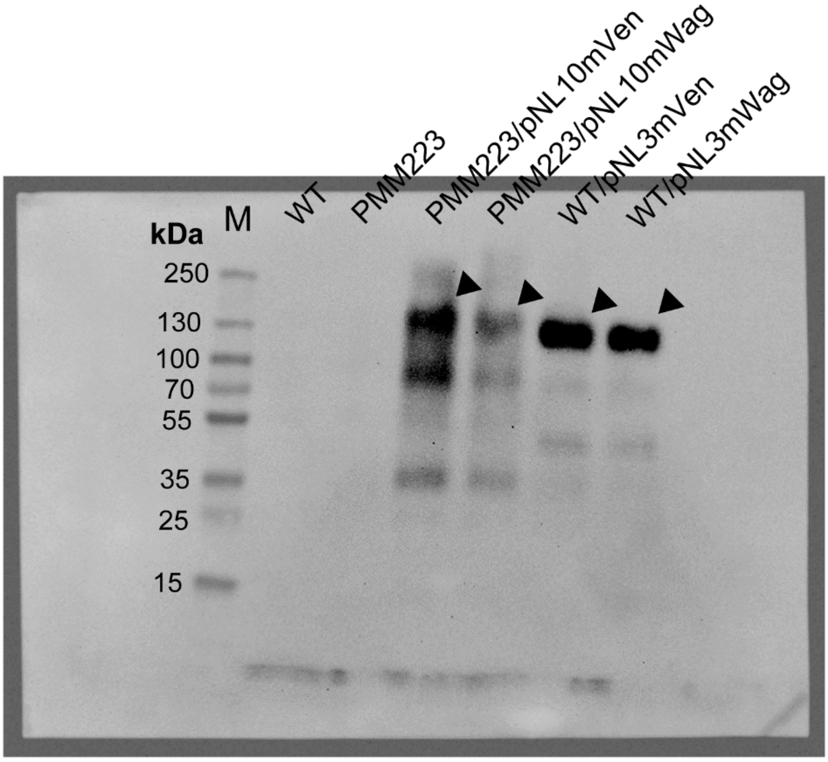

Supplementary Figure S2. Western blotting analysis of total cell lysates from strains used in this study. Whole-cell lysates were prepared as described previously[1], separated by SDS-PAGE and transferred onto nitrocellulose membranes (Pall Corp.). Membranes were incubated in 1X PBS containing 0.1% Tween 20 and 10% nonfat dry milk and then incubated for 1 h with a mouse anti-eGFP monoclonal antibody (ThermoFisher, 1/1000) in PBS containing 0.1% Tween 20 and 1% dry milk. Membranes were washed three times for 5 min with PBS containing 0.1% Tween 20 and incubated for 1 h with a horseradish peroxidase–conjugated goat anti-mouse IgG antibody (Sigma, 1/3000) in PBS containing 0.1% Tween 20 and 1% nonfat dry milk. Protein bands were visualized using the EMD Millipore Immobilon enhanced chemiluminescence kit (Fisher Scientific) and a ChemiDoc Touch imaging system (Bio-Rad). Migratory positions of full-length MmpL10-mVenus (calculated molecular mass 134.3 kD) and MmpL3-mVenus (calculated molecular mass 137.3 kD) fusion proteins are indicated by arrowheads. Of note, despite a higher calculated molecular mass, MmpL3-mVenus migrates faster in SDS-PAGE than MmpL10-mVenus. M: protein molecular weight marker (PageRuler Plus Stained Protein Ladder, ThermoFisher Scientific).

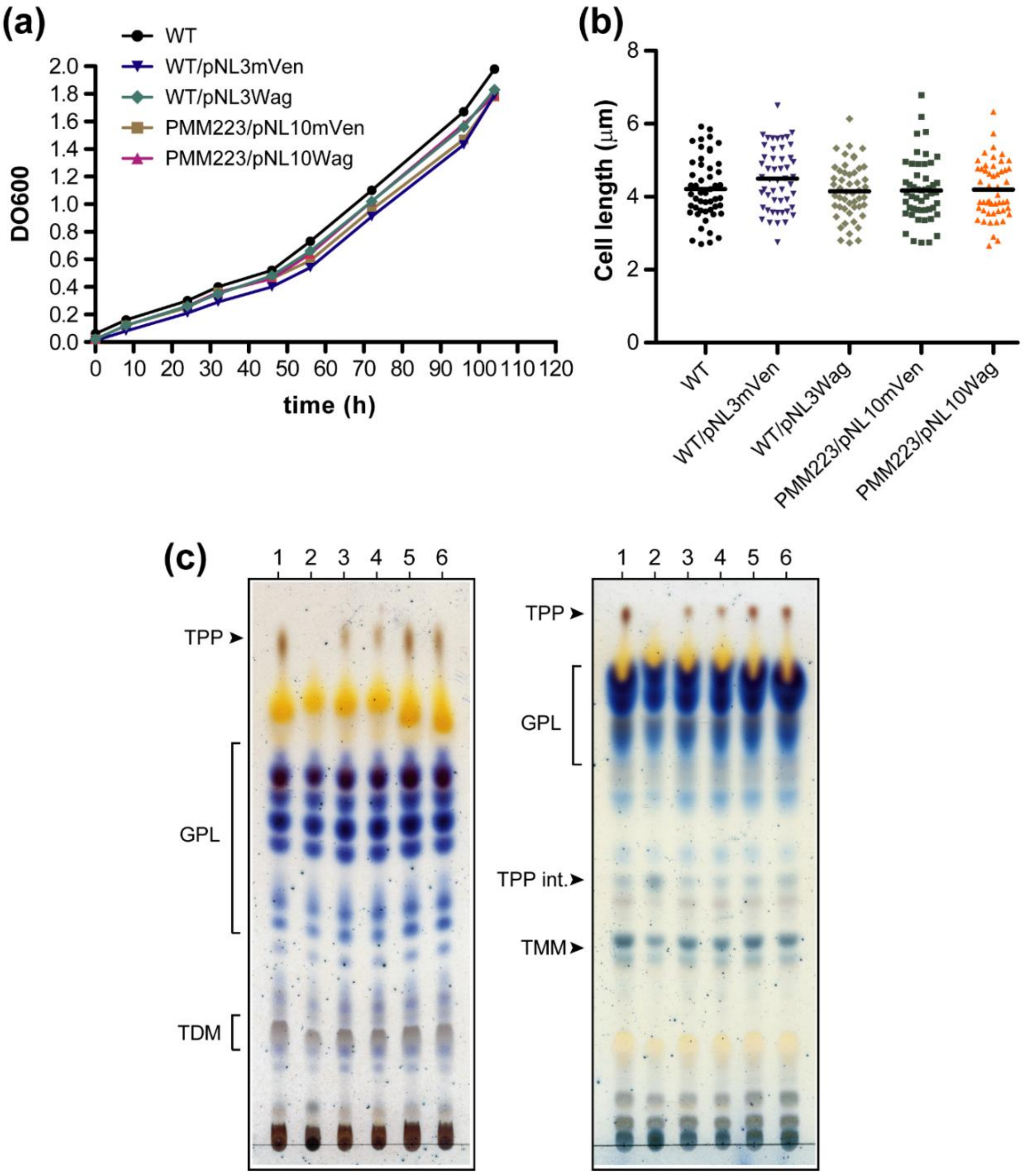

Supplementary Figure S3. Growth curves, cell length measurements and lipid profiles of strains used in this study. (**a**) Growth kinetics of bacterial strains were monitored by measuring the optical density (OD_{600}) over time of bacterial cultures grown in 7H9/Tween 80 with or without hygromycin, inoculated at an initial DO_{600} of 0.05. (**b**) The average cell length of each strain was determined by labeling 50 randomly selected individual bacteria, without visible septum, with HADA for 20 min. Bacteria were then washed and their length measured via microscopy. (**c**) Lipid profiles of the *M. smegmatis* WT strain (1) and of the PMM223 (2), PMM223/pNL10mVen (3), PMM223/pNL10Wag (4), WT/pNL3mVen (5), WT/pNL3Wag (6) strains. Bacteria were cultivated in 7H9 with or without hygromycin. Total lipid fractions were extracted as described elsewhere[1] and loaded onto TLC plates run in $CHCl_3/CH_3OH$ (90:10, v/v) (left) or $CHCl_3/CH_3OH/H_2O$ (60:16:2, v/v) (right). The spots were visualized by spraying TLC plates with 0.2% (w/v) anthrone in concentrated H_2SO_4, followed by heating. Positions of trehalose polyphleates (TPP), diacyltrehalose TPP intermediates (TPP int.)[2], glycopeptidolipids (GPL), trehalose monomycolate (TMM), and trehalose dimycolates (TDM) are indicated.

Supplementary Table S1: Oligonucleotides used in this study.

Primer	Oligonucleotide sequence (5’–3’)
16165a	ATACAACATATGCGTAAGCTAGCCGATCTTG
16165c	ACAAAAGCTTGGTCACCTCTGCCGGCGCC
17331a	AGAGGTGACCAAGCTTAGTGGAGCGGCCGCTGCAG
17331b	ACTACGTCGACATCGATGTTTAAACTCACTTGTACAGCTCG
18075d	GAAATCTAGATTAATTAAGCCGTTGTCCTCCCTAGAATC
18075e	GCTAGCTTACGCATATGACGTGGTCCTTCCCAATAATTC
19170a	ATAACATATGTTCGCCTGGTGGGGTCG
19170b	ATAAAAGCTTCAGCCTGCCTTCGCGGCG
19318a	AACGGCTTAATTAATCTAGATTCATCGGCATGCAGCTCGC
19318b	GTTCCCGCCAGAAATCTAGATTACTTATAGAGCTCGTCCATCC
23016a	CCCTGTGTCGAATTTCTTCCAC
23016b	ACGTTCCAGGAACGCCACCG
23016c	GAGGAACACCATGGACTGATTG
res1	GCTCTAGAGCAACCGTCCGAAATATTATAAA
res2	GCTCTAGATCTCATAAAAATGTATCCTAAATCAAATATC

Supplementary References

1 Thouvenel, L. *et al.* The final assembly of trehalose polyphleates takes place within the outer layer of the mycobacterial cell envelope. *J Biol Chem* **295**, 11184-11194, doi:10.1074/jbc.RA120.013299 (2020).

2 Burbaud, S. *et al.* Trehalose Polyphleates Are Produced by a Glycolipid Biosynthetic Pathway Conserved across Phylogenetically Distant Mycobacteria. *Cell Chem Biol* **23**, 278-289, doi:10.1016/j.chembiol.2015.11.013 (2016).
